# 1824. Impact of Social Determinants of Health on Preferred Treatment of *Trichomonas vaginalis* and *Chlamydia trachomatis*

**DOI:** 10.1093/ofid/ofad500.1653

**Published:** 2023-11-27

**Authors:** Christen Arena, Rachel M Kenney, Erin Eriksson, Indira Brar, Michael Veve

**Affiliations:** Eugene Applebaum College of Pharmacy and Health Sciences/Wayne State University/Henry Ford Hospital, Royal Oak, Michigan; Henry Ford Hospital, Detroit, Michigan; Henry Ford Health, Detroit, Michigan; Henry Ford Hospital, Detroit, Michigan; Henry Ford Health, Detroit, Michigan

## Abstract

**Background:**

The 2021 CDC Sexually Transmitted Infections (STI) Treatment Guideline modified preferred therapy for *Trichomonas vaginalis* (TV) and *Chlamydia trachomatis* (CT) from single dose to a 7-day course. Social Determinants of Health (SDOH) are non-medical factors that influence a person’s life; little is known regarding the association between SDOH and TV and CT treatment. The study objective was to evaluate treatment of TV and CT infections after the guideline update and determine if health inequities exist with use of preferred therapy.

**Methods:**

IRB approved, retrospective cohort of patients ≥ 15 years with confirmed diagnosis of uncomplicated TV or CT in outpatient settings. Excluded: pregnant/nursing, allergy to preferred therapy, unable to take oral. Primary outcome: proportion who received guideline preferred vs non-preferred (alternative, discordant, or null) antibiotic therapy. Logistic regression was used to identify variables associated with preferred treatment; SDOH were exposures of interest. Secondary outcomes: test of cure (≤ 3 months), any repeat positive test (recurrence/reinfection), any retreatment, expedited partner therapy (EPT) offered. A sample of 712 patients was needed to detect a 10% difference between two exposures (α=0.05, β=0.2).

**Results:**

473 (66%) patients received preferred therapy; patient characteristics are in **Table 1.** Patients < 25 years had more asymptomatic disease compared to older patients (198 [54%] vs 150 [44%], *P*=0.01). Patients who received Emergency Department (ED) care were more likely to receive preferred therapy compared to outpatient clinics (201/264 [76%] vs 272/448 [61%], *P*=< 0.001). Black race, lower median income, and public insurance covaried with ED care. After adjusting for female sex, receipt of ED care was independently associated with preferred therapy (**Table 2**). 181 (25%) patients had 3-month test of cure performed; repeat positive test/retreatment was more frequent in patients who received non-preferred therapy (25 [11%] vs 24 [5%], *P*=0.01). EPT was offered in 35 (7%) and 8 (3%) patients in the preferred and non-preferred groups (*P*=0.03).

**Figure:**
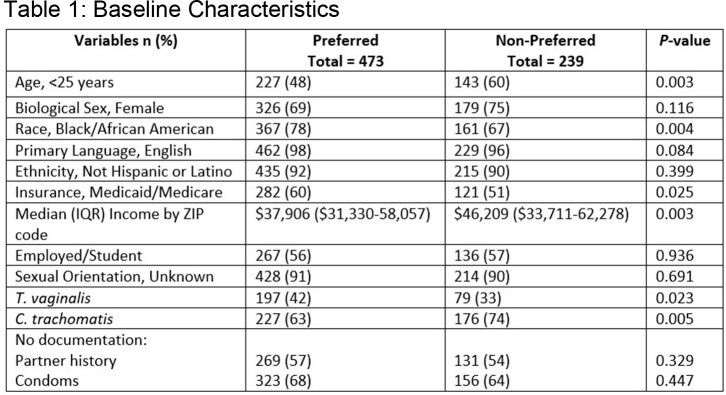

**Table 2:**
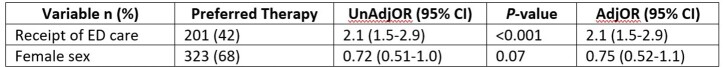
Variables Associated with Receipt of Preferred Therapy. Abbreviations: ED, Emergency Department; UnAdjOR, unadjusted odds ratio; AdjOR, adjusted odds ratio

**Conclusion:**

Preferred therapy was more frequent in patients who received ED care. EDs represent an important safety net and provide high-level care for patients with SDOH barriers.

**Disclosures:**

**Erin Eriksson, PharmD, BCPS, BCIDP**, Stryker Corp: Stocks/Bonds **Indira Brar, MD**, Gilead: Advisor/Consultant|Gilead: Grant/Research Support|Gilead: Honoraria|Janssen: Grant/Research Support|Janssen: Honoraria|ViiV: Advisor/Consultant|ViiV: Grant/Research Support|ViiV: Honoraria **Michael Veve, PharmD, MPH**, National Institutes of Health: Grant/Research Support|Paratek Pharmaceuticals: Grant/Research Support

